# Georeferencing of Laser Scanner-Based Kinematic Multi-Sensor Systems in the Context of Iterated Extended Kalman Filters Using Geometrical Constraints

**DOI:** 10.3390/s19102280

**Published:** 2019-05-17

**Authors:** Sören Vogel, Hamza Alkhatib, Johannes Bureick, Rozhin Moftizadeh, Ingo Neumann

**Affiliations:** Geodetic Institute, Leibniz Universität Hannover, Nienburger Str. 1, 30167 Hannover, Germany; alkhatib@gih.uni-hannover.de (H.A.); bureick@gih.uni-hannover.de (J.B.); rozhin.moftizadeh@stud.uni-hannover.de (R.M.); neumann@gih.uni-hannover.de (I.N.)

**Keywords:** georeferencing, kinematic multi-sensor system, implicit model, iterated extended Kalman filter, inequality state constraints, probability density function truncation

## Abstract

Georeferencing is an indispensable necessity regarding operating with kinematic multi-sensor systems (MSS) in various indoor and outdoor areas. Information from object space combined with various types of prior information (e.g., geometrical constraints) are beneficial especially in challenging environments where common solutions for pose estimation (e.g., global navigation satellite system or external tracking by a total station) are inapplicable, unreliable or inaccurate. Consequently, an iterated extended Kalman filter is used and a general georeferencing approach by means of recursive state estimation is introduced. This approach is open to several types of observation inputs and can deal with (non)linear systems and measurement models. The capability of using both explicit and implicit formulations of the relation between states and observations, and the consideration of (non)linear equality and inequality state constraints is a special feature. The framework presented is evaluated by an indoor kinematic MSS based on a terrestrial laser scanner. The focus here is on the impact of several different combinations of applied state constraints and the dependencies of two classes of inertial measurement units (IMU). The results presented are based on real measurement data combined with simulated IMU measurements.

## 1. Introduction

Multi-sensor systems (MSS) are greatly used nowadays in geodesy to capture an environment for various applications. Georeferencing is required in most cases for these data to be applicable. In simple and straightforward words, georeferencing is to derive the position and orientation of a platform with respect to a superordinate coordinate system. Therefore, in a static case, georeferencing would mean to derive six pose parameters (three translations and three rotations), whereas for kinematic platforms, the six degrees of freedom (DOF) should be calculated separately for each time epoch [1]. In general, there is no need to consider a scale factor as additional DOF, as long as sensors (e.g., laser scanner) of the MSS are consistent with each other during data acquisition. If there is a necessity, extension to a three-dimensional (3D) similarity transformation can be applied [2].

This indispensable necessity of precise and accurate pose parameters is a frequent challenge for outdoor and indoor mapping applications. Depending on respective complex or challenging environments common methods for georeferencing might fail, are unreliable or are at least inaccurate. The main reasons for this are missing or inaccurate observations from a global navigation satellite system (GNSS) within indoor spaces or inner-city environments caused by shadowing or multipath effects. In addition, further methods (e.g., visual odometry) and sensors (e.g., inertial measurement unit (IMU)) have to deal with significant drifts in pose estimation [1,3,4,5]. In order to improve georeferencing for such challenging circumstances, a Kalman filter-based approach is extended and validated by means of a laser scanner-based kinematic MSS within this paper. As a novelty, arbitrary explicit and implicit measurement equations as well as nonlinear equality and inequality state constraints can be applied.

### 1.1. Georeferencing of Kinematic Multi-Sensor Systems

Georeferencing is generally realized through three different approaches, each of them deals with various methods that are based on the sensors available and environmental conditions. These approaches are called direct, indirect and data-driven georeferencing [2]. In direct georeferencing, position and orientation of a measuring platform are derived directly from the sensors available on board, such as a GNSS antenna [2], an IMU or an external sensor, such as a laser tracker [6] or a total station [4]. However, this approach depends highly on the environmental circumstances (e.g., visibility in complex indoor interior or absence of GNSS observations). In indirect georeferencing, observations of other sensors available on the platform, such as laser scanners or cameras are taken into account. In this approach, common environmental information (e.g., known control points for laser scanners by means of artificial targets) which are captured both in the local sensors’ coordinate system and in a superordinate coordinate system are linked together [7]. Approaches for data-driven georeferencing require point cloud information which has already been georeferenced. This can be given by means of 3D city models, floor plans or other maps of the environment requested. Several arbitrary matching algorithms can be applied to get the position and orientation of an MSS which is acquiring point cloud data regarding models or maps mentioned. Uncertainty of the prior information affects the final georeferencing solution significantly. Known approaches for this method generally rely on iterative closest point (ICP) algorithms or rather on simultaneous localization and mapping (SLAM) methods [5,8,9,10,11].

### 1.2. Kalman Filter Techniques for Georeferencing

Combinations of aforementioned approaches are also possible and advisable to increase the accuracy and precision of the georeferencing of a kinematic MSS. This data fusion is commonly covered within the system state of a filtering approach. Such recursive approaches enable possibilities to handle big data, which come along with present and future multi-sensor technologies. Furthermore, they are suitable for online applications and usually require less memory and computational effort than batch algorithms that have been adapted for online georeferencing applications [12].

A Kalman filter (KF) is a well-known two-step procedure for this in which the next system state is estimated based on the previous state information and recent observations subsequently. Therefore, an iterative process is required by utilizing nonlinear measurement equations, which seem to be the most logical choice in case of trajectories. Consequently, such a procedure is called iterated extended Kalman filter (IEKF). A standard extended Kalman filter (EKF) can also handle nonlinear equations. However, an IEKF with further iterations is more suitable in the case of high nonlinear functions and will provide more accurate results using only small additional computational effort. Handling with nonlinear equations can also be done by means of unscented transformation (UT) as part of the unscented Kalman filter (UKF).

So far, in almost every research only explicit measurement equations are considered for such filter approaches. This means that the observations are taken into account as a function of the state parameters. Such a model is generally referred to a Gauss–Markov model (GMM). However, the use of a Gauss–Helmert model (GHM), which gives the possibility to implicitly link the observations to the state parameters, has also been studied by a few researchers [13,14,15,16,17]. Such a methodology provides the opportunity to include all kinds of measurement equations into the filtering approach, regardless whether they are of an implicit or explicit nature. A basic algorithm for nonlinear implicit measurement equations within an IEKF for extrinsic auto-calibration of a stereo rig is proposed in [13,15]. The algorithm is used for the extrinsic auto-calibration of a stereo rig which has led to satisfying results. Furthermore, a linear KF with respect to GHM is developed in [17] and applied for orientation determination with smartphone sensors. However, both contributions do not consider state constraints. In addition, the latter is based on a linear KF approach. In [14], implicit measurement equations within a recursive estimation approach for Kalman filtering are referred to as implicit constraints. Usage of implicit measurement equations in terms of an UKF does not exist at all.

### 1.3. Contribution

Except for [16], the approaches mentioned within Section 1.2 have neglecting additional state constraints in common. Although, it is very useful to consider suitable environmental scene information by means of equality or inequality state constraints. This possibility is frequently used and evidenced in terms of well-known filter approaches with explicit formulations [2,18,19,20]. Horizontal and vertical lines, parallel or perpendicular lines and different planes in a scene are examples of such information which could be used as assigned geometric constraints during data analysis. In the recent work by [16], an IEKF by means of implicit measurement equations and nonlinear equality constraints is used for georeferencing of a simulated kinematic MSS. As a novelty, this approach is extended by nonlinear inequality state constraints within this paper. This increases the possibilities to apply any suitable geometrical prior information and to improve the georeferencing solution even in such challenging environments mentioned. Fundamental applicability is shown by means of a real kinematic MSS within an indoor environment and validated by highly accurate reference information. Additionally, a more general overview of the filter approach is given within this paper to make the approach independent from specific MSS, environments and prior information used.

### 1.4. Outline

The dedicated sections of this paper are as follows. An overview of the general georeferencing approach by means of a recursive state estimation is introduced in Section 2. This algorithm proposed is confirmed by being applied to a real data-set of an indoor environment that is captured by a kinematic MSS equipped with a TLS and tracked by a laser tracker in Section 3. The paper ends with a discussion of the results presented in Section 4 whereas Section 5 concludes this contribution.

## 2. General Georeferencing Approach by Means of Recursive State Estimation

A standardized estimation approach is indispensable to ensure an accurate, precise, reliable and complete georeferencing solution of different arbitrary kinematic MSS. The drawbacks mentioned in Section 1 could be eliminated only by providing a generally valid framework, which is applicable to as many use cases and systems as possible. For this reason, a recursive state estimation approach, which is compatible with various types of input data (e.g., requested states, available sensor observations and additional prior information), is formulated in this paper. However, the basic structure and equations used are with respect to [16]. The carefully selected information depends on each individual application and its respective circumstances. However, they are combined and fused in a unified way within the general valid framework to deliver optimal results. Necessary demands on the input data can be divided into four interconnected questions:

- Which types of sensor observations (e.g., laser scanner, GNSS, IMU, total station) are available and what are their accuracies?
- Which suitable and reliable prior information (e.g., geometrical circumstances, landmarks, maps) are available?
- What is the mathematical relationship between all input data?
- What information about the physical model of the system is known?

**Figure 1:**
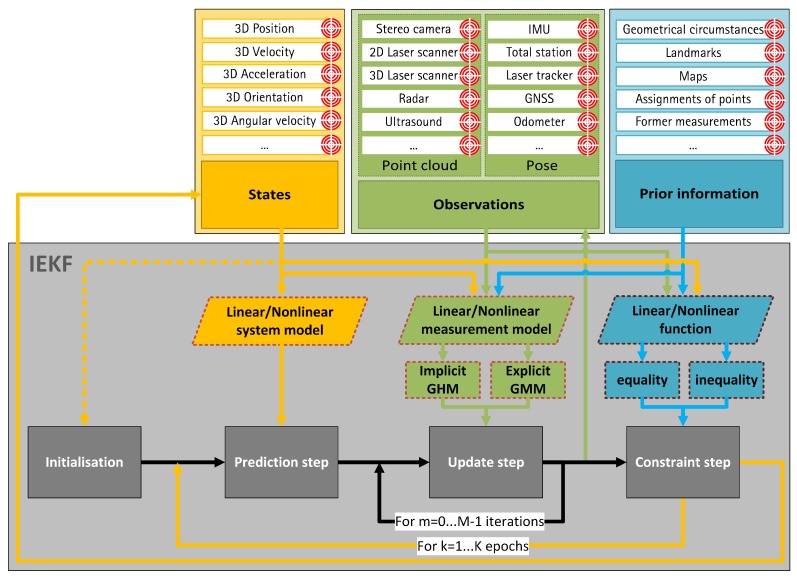
Schematic procedure of the universal recursive filter approach for georeferencing of a kinematic multi-sensor systems (MSS). Steps of the iterated extended Kalman filter (IEKF) (grey) are depicted with possible requested states (yellow), available observations (green) and known prior information (blue). Respective uncertainty information are depicted by red target circles.

In theory, all possible input data should be considered. These input possibilities are restricted to the most common ones for the sake of simplification and according to the current paper perspectives. However, it should be noted that other types of input data are also possible and should be considered based on the application. A schematic overview of the universal recursive filter approach for georeferencing of a kinematic MSS together with corresponding relations between states, observations, prior information and respective parts of an IEKF is illustrated in Figure 1. In addition to the input data (states and observations) and prior information, sets of fundamental functions have to be formulated and integrated into the process. An arbitrary system model will describe the physical behaviour of the MSS between neighbouring epochs. Any model from the current state of the art can be selected for this. Total neglection of the system model is also possible and will result in a sequential adjustment approach. Formulation of a measurement model can happen in an implicit and/or explicit manner. (Non)linear functions regarding the states can also be added by means of equality and/or inequality formulations. Such state constraints can be integrated by means of several different methods (e.g., pseudo observations, projection method, probability density function (PDF) truncation or soft constraints). However, equality and inequality constraints have the crucial advantage of including specific further information into the filter approach by means of clear values (in the case of equalities) or thresholds (in the case of inequalities).

### 2.1. Iterated Extended Kalman Filter with Nonlinear Implicit Measurement Equation

The basic structure of the georeferencing approach applied is based on the IEKF, which was published by [13] and enables the possibility of integrating implicit measurement equations (of type h(l,x)=0) within the recursive estimation process. This gives the possibility to consider inextricable relationships between states x and observations l within the observation model. Only explicit relationships (of type l=h(x)) are allowed within normal KF, which results in major restrictions. As shown in Section 3, there is an important demand for implicit equations within the IEKF process in order to consider more challenging relationships. However, explicit measurement equations are still possible to use but will be converted into l−h(x)=0 to fulfil the implicit statement.

There are nonlinear measurement equations h(·) within the IEKF which provide a connection between the observations measured and states requested. The physical behaviour of the MSS over time is formulated within the nonlinear system model f(·) over all epochs k=1,…,K theoretically:

(1) $$\begin{array}{c} h\left(l_{k}+v_{k},x_{k}\right)=0,\;\;\;v_{k}∼N\left(0,\Sigma_{\mathrm{vv}}\right) \end{array}$$

(2) $$\begin{array}{c} x_{k}=f\left(x_{k-1},u_{k-1},w_{k-1}\right),\;\;\;w_{k-1}∼N\left(0,\Sigma_{\mathrm{ww}}\right). \end{array}$$

Here, u is the deterministic control by means of external controls. The variance-covariance matrix (VCM) $\Sigma_{\mathrm{ww}}$ of the system noise w and $\Sigma_{\mathrm{vv}}$ of the measurement noise v are normally distributed with zero mean. Regarding our universal recursive filter approach, the IEKF is divided into a prediction step using the system model, an update step making use of the measurement model and into a constrained step for applying known prior information by means of linear $Dx_{k}$ and/or nonlinear $g\left(x_{k}\right)$ state constraints. All three steps are described within [16] in detail and are summarized in Algorithm 1.

**Algorithm 1:** Iterated extended Kalman filter (IEKF) with nonlinear implicit measurement equation and nonlinear equality state constraints.

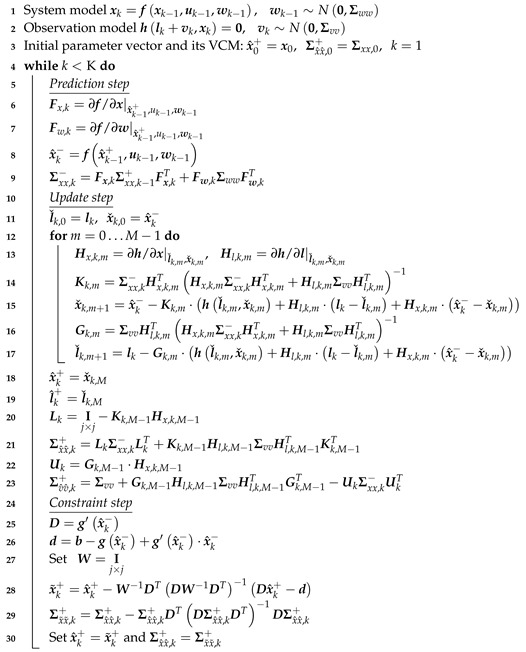



It is worth mentioning that up to now, to the best of our knowledge, no research which deals with state constraints in connection with implicit measurement equations are being investigated. All published methods are regarding explicit measurement equations where it is possible to separate states and observations from each other. However, in terms of implicit measurement equations of type h(l,x)=0, modified state parameters during the constraint step will violate this equation because of the unaffected observations during the constraint step. This fact is currently being dealt with by the authors and is under investigation. However, our results in Section 3 show the fundamental validity.

### 2.2. Inequality State Constraints by Means of Probability Density Function Truncation

The constrained step within this paper is extended to allow the possibility of considering inequality state constraints. Instead of using the projection method (cf. Algorithm 1, line 28–30), the flexible PDF truncation method is used, and both given in [18] and [20]. In theory, other methods also mentioned above (e.g., pseudo observations) can be applied in order to consider state constraints. However, usage of state constraints in combination with implicit measurement equations is so far not considered for any Kalman filtering technique. By using the PDF truncation, equality and inequality constraints can be included simultaneously by the same method and there is no need to perform inefficient quadratic programming techniques. Furthermore, numerical instabilities resulting from e.g., singular measurement noise covariance in the context of perfect measurements can be avoided [19]. Depending on the respective conditions, the thresholds could be set by means of lower ${\mathrm{lb}}_{i,k}$ and upper ${\mathrm{ub}}_{i,k}$ boundaries for s scalar two-sided state constraints for any arbitrary nonlinear functions $g_{i}$ of the states.

(3) $$\begin{array}{c} {\mathrm{lb}}_{i,k}\leq g_{i}\left(x_{k}\right)\leq {\mathrm{ub}}_{i,k}\;\;\;\;\;\;\;\;\;\;\;\;\;\;i=1,\ldots ,s. \end{array}$$

Within this PDF truncation method, the estimated PDF of the IEKF (assumed in this paper as Gaussian) is truncated by means of the defined lower and upper boundaries and, subsequently, recomputed to the constrained estimate at the mean of the truncated PDF. Realization of the PDF truncation method is carried out for every single constraint i=1…s successively. Furthermore, ${\tilde{x}}_{i,k}$ will be the state estimate after applying the i-th constraint and ${\tilde{\Sigma }}_{i,k}$ will be its respective VCM. Their initialization for i=0 is achieved by updated KF estimations ${\tilde{x}}_{0,k}={\hat{x}}_{k}^{+}$ and ${\tilde{\Sigma }}_{0,k}=\Sigma_{\hat{x}\hat{x},k}^{+}$. Afterwards, a transformation from $x_{i,k}$ to $z_{i,k}$ is performed for the decoupling of the s constraints:

(4) $$\begin{array}{c} z_{i,k}=S_{i}\cdot W_{i}^{-\frac{1}{2}}\cdot T_{i}^{T}\left(x_{k}-{\tilde{x}}_{i,k}\right). \end{array}$$

The diagonal matrices $W_{i}$ and orthogonal matrices $T_{i}$ are obtained by performing Jordan canonical decomposition of the VCM ${\tilde{\Sigma }}_{i,k}$:

(5) $$\begin{array}{c} T_{i}\cdot W_{i}\cdot T_{i}^{T}={\tilde{\Sigma }}_{i,k}. \end{array}$$

The orthogonal matrix $S_{i}$ is determined by using Gram–Schmidt orthogonalization (cf. [18]) and satisfies:

(6) $$\begin{array}{c} S_{i}\cdot W_{i}^{-\frac{1}{2}}\cdot T_{i}^{T}\cdot g_{i}\left(x_{k}\right)={\left[\begin{array}{cccc} {\left(g_{i}{\left(x_{k}\right)}^{T}\cdot {\tilde{\Sigma }}_{i,k}\cdot g_{i}\left(x_{k}\right)\right)}^{\frac{1}{2}} & 0 & \ldots & 0 \end{array}\right]}^{T}. \end{array}$$

The normalized scalar constraint could be derived using this transformation, where $z_{i,k}$ has a zero mean and a VCM of identity.

(7) $$\begin{array}{c} a_{i,k}\leq \left[\begin{array}{cccc} 1 & 0 & \ldots & 0 \end{array}\right]\cdot z_{i,k}\leq b_{i,k}. \end{array}$$

The transformed boundaries $a_{i,k}$ and $b_{i,k}$ are:

(8) $$\begin{array}{c} a_{i,k}=\frac{{\mathrm{lb}}_{i,k}-g_{i}\left(x_{k}\right)\cdot {\tilde{x}}_{i,k}}{{\left(g_{i}{\left(x_{k}\right)}^{T}\cdot {\tilde{\Sigma }}_{i,k}\cdot g_{i}\left(x_{k}\right)\right)}^{\frac{1}{2}}}\;,\;\;\;\;\;\;\;b_{i,k}=\frac{{\mathrm{ub}}_{i,k}-g_{i}\left(x_{k}\right)\cdot {\tilde{x}}_{i,k}}{{\left(g_{i}{\left(x_{k}\right)}^{T}\cdot {\tilde{\Sigma }}_{i,k}\cdot g_{i}\left(x_{k}\right)\right)}^{\frac{1}{2}}}. \end{array}$$

Truncation of the Gaussian PDF by means of the lower an upper bound and the integration variables ζ and γ is implemented by:

(9) $$\begin{array}{c} \int_{a_{i,k}}^{b_{i,k}}\frac{1}{\sqrt{2\pi }}\cdot e^{-\frac{\zeta^{2}}{2}}d\zeta =\frac{1}{2}\left[\mathrm{erf}\left(\frac{b_{i,k}}{\sqrt{2}}\right)-\mathrm{erf}\left(\frac{a_{i,k}}{\sqrt{2}}\right)\right]\;,\;\;\;\mathrm{erf}\left(u\right)=\frac{2}{\sqrt{\pi }}\int_{0}^{u}e^{-\gamma^{2}}d\gamma . \end{array}$$

The normalized truncated PDF within the boundaries $a_{i,k}$ and $b_{i,k}$ is given by:

(10) $$\begin{array}{c} \mathrm{pdf}\left(\zeta \right)=\beta_{i,k}\cdot e^{\frac{-\zeta^{2}}{2}}\;,\;\;\;\;\;\beta_{i,k}=\frac{\sqrt{2}}{\sqrt{\pi }\cdot \left[\mathrm{erf}\left(\frac{b_{i,k}}{\sqrt{2}}\right)-\mathrm{erf}\left(\frac{a_{i,k}}{\sqrt{2}}\right)\right]}. \end{array}$$

The mean $\mu_{i,k}$ and variance $\sigma_{i,k}^{2}$ of the i-th element of $z_{i,k}$ is computed by:

(11) $$\begin{array}{cc} \mu_{i,k} & =\beta_{i,k}\cdot \left[e^{\frac{-a_{i,k}^{2}}{2}}-e^{\frac{-b_{i,k}^{2}}{2}}\right] \end{array}$$

(12) $$\begin{array}{cc} \sigma_{i,k}^{2} & =\beta_{i,k}\cdot \left[e^{\frac{-a_{i,k}^{2}}{2}}\cdot \left(a_{i,k}-2\cdot \mu_{i,k}\right)-e^{\frac{-b_{i,k}^{2}}{2}}\cdot \left(b_{i,k}-2\cdot \mu_{i,k}\right)\right]+\mu_{i,k}^{2}+1. \end{array}$$

With this, the mean ${\tilde{z}}_{i+1,k}$ and VCM ${\tilde{C}}_{i+1,k}$ of the transformed state could be estimated:

(13) $$\begin{array}{cc} {\tilde{z}}_{i+1,k} & ={\left[\begin{array}{cccc} \mu_{i,k} & 0 & \ldots & 0 \end{array}\right]}^{T} \end{array}$$

(14) $$\begin{array}{cc} {\tilde{C}}_{i+1,k} & =\mathrm{diag}\left(\sigma_{i,k}^{2},1,\ldots ,1\right). \end{array}$$

The ${\tilde{x}}_{i+1,k}$ and its corresponding VCM ${\tilde{\Sigma }}_{i+1,k}$ of the state are estimated by means of inversion of transformation in (4):

(15) $$\begin{array}{cc} {\tilde{x}}_{i+1,k} & =T_{i}\cdot W_{i}^{-\frac{1}{2}}\cdot S_{i}^{T}\cdot {\tilde{z}}_{i+1,k}+{\tilde{x}}_{i,k} \end{array}$$

(16) $$\begin{array}{cc} {\tilde{\Sigma }}_{i+1,k} & =T_{i}\cdot W_{i}^{-\frac{1}{2}}\cdot S_{i}^{T}\cdot {\tilde{C}}_{i+1,k}\cdot S_{i}\cdot W_{i}^{-\frac{1}{2}}\cdot T_{i}^{T}. \end{array}$$

Finally, after performing this for all *s* constraints in series, the constrained states ${\tilde{x}}_{k}$ and their VCM ${\tilde{\Sigma }}_{k}$ could be derived as:

(17) $$\begin{array}{cc} {\tilde{x}}_{k} & ={\tilde{x}}_{s,k} \end{array}$$

(18) $$\begin{array}{cc} {\tilde{\Sigma }}_{k} & ={\tilde{\Sigma }}_{s,k}. \end{array}$$

The whole procedure of PDF truncation for involving inequality constraints is depicted in Algorithm 2. In order to handle one-sided inequality constraints, ${\mathrm{lb}}_{i,k}=-\infty$ or ${\mathrm{ub}}_{i,k}=\infty$ could be used. In the case of equality constraints, Equations (7), (8), (11), (12) are required to be changed to perform PDF truncation:

(19) $$\begin{array}{cc} c_{i,k} & =\left[\begin{array}{cccc} 1 & 0 & \ldots & 0 \end{array}\right]\cdot z_{i,k} \end{array}$$

(20) $$\begin{array}{cc} c_{i,k} & =\frac{d_{i,k}-g_{i}\left(x_{k}\right)\cdot {\tilde{x}}_{i,k}}{{\left(g_{i}{\left(x_{k}\right)}^{T}\cdot {\tilde{\Sigma }}_{i,k}\cdot g_{i}\left(x_{k}\right)\right)}^{\frac{1}{2}}} \end{array}$$

(21) $$\begin{array}{cc} \mu_{i,k} & =c_{i,k} \end{array}$$

(22) $$\begin{array}{cc} \sigma_{i,k}^{2} & =0. \end{array}$$

**Algorithm 2:** Probability density function (PDF) truncation for inequality state constraints.

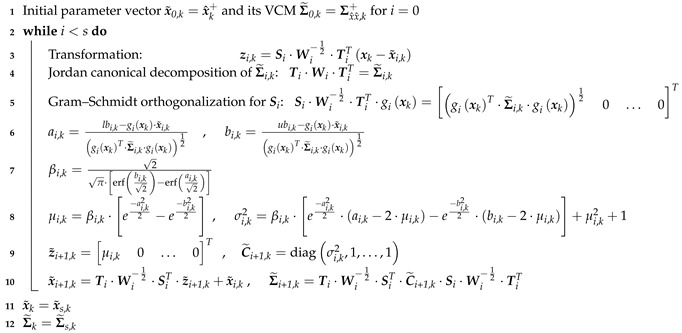



## 3. Application in Terms of Accurate Indoor Georeferencing of a k-TLS

Various MSS in terms of calibration, acquisition and georeferencing are developed and used in practice at the Geodetic Institute Hannover (GIH) of the Leibniz University Hannover. Within this case study, the proposed general georeferencing approach from Section 2 is applied to a kinematic MSS extensively described in [6,21]. Utilizing such a proved MSS allows us to focus on the application of the proposed theoretical approach and to rely on an already calibrated and synchronized system. Furthermore, highly accurate validation by means of a laser tracker is possible. Such a comparison based on the trajectory is much more accurate than based on the 3D point cloud (e.g., TLS targets).

### 3.1. Overview

**Figure 2:**
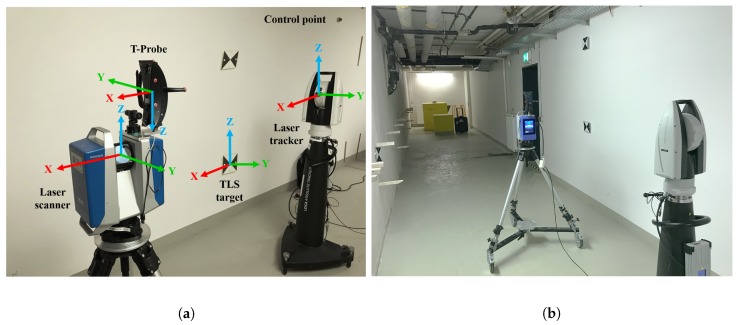
A general view of the kinematic MSS with its coordinate systems (**a**) used in the basement of the Geodetic Institute Hannover (GIH) (**b**).

The kinematic MSS consists of a 3D TLS, a laser tracker, and a special probe (a normally hand-held combination of a reflector and ten LEDs for pose estimation regarding the laser tracker). The TLS is a Zoller + Fröhlich Imager 5016, which is used in a 2D profile mode for this application. The measuring rate of this sensor is 55 profiles/second and its range noise is 0.3 mm for a distance of 10 m [22]. The TLS is mounted on a rollable platform. On the other hand, the laser tracker used is a Leica AT960 LR with its Leica T-Probe. The T-Probe is rigidly mounted on top of the TLS and, therefore, moves with the TLS along the trajectory (cf. Figure 2a). Combination of the laser tracker with the T-probe gives the position with an accuracy of ±15 μm + 6 μm/m (as a maximum permissible error (MPE) for the 3D position) and the orientation with an MPE of $0.01^{\circ }$ = 18 μm/100 mm for the accuracy of each orientation-direction, respectively [23]. Due to the integration of such a highly accurate laser tracker into this MSS, the reference pose information with superordinated accuracy could be derived directly.

**Figure 3:**
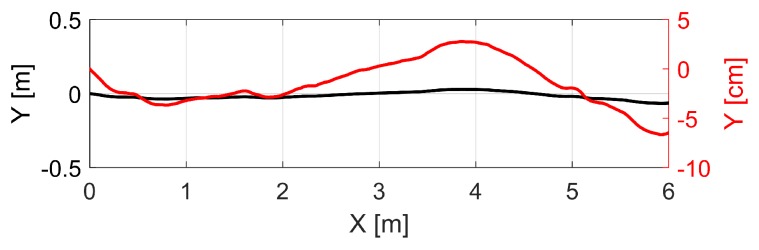
Top view of the measured trajectory obtained by the laser tracker. Two visualizations of the same trajectory in order to highlight the almost linear course. The black curve is regarding the left y-axis (meter) and the red curve regarding the right y-axis (centimetre).

Data acquisition for this case study was carried out within the basement of the GIH inside a selected section of a corridor (cf. Figure 2b). Characteristics of this section are ideal with extensive walls and obstacles such as pipes below the ceiling and a door in one wall. The kinematic MSS was moved through this environment on an almost linear six meter long trajectory for about 25 s at a slow walking speed. This corresponds to 1311 epochs in total. We were aware that the used trajectory was limited in time and space. However, we intended here only to focus on the applicability of our developed georeferencing approach. The laser tracker was referenced in advance by given control points to ensure transformation to a superordinated coordinate system. The TLS targets regarding this coordinate system are also provided inside the environment measured. They will support further validation of our approach based on 3D point cloud information in the future. However, this issue was not in the focus of this paper. Instead, we will use the highly accurate reference pose by means of the laser tracker for validation. The TLS captured 3D points in a profile mode regarding its local sensor coordinate system. Every full laser scanner profile (LSP) was linked to 6D pose information by means of the laser tracker and T-probe. The right geometrical relation of this 6D pose to the reference point of the TLS was done by means of given calibration parameters (cf. [6]). The kinematic MSS utilized together with all the coordinate systems mentioned are depicted in Figure 2a. Thus, the direction of movement of the MSS is in x-direction. Consequently, the LSPs captured were in the “y–z” plane of the local laser scanner coordinate system. A highly accurate static full 3D laser scan of the captured section of the environment by means of the same laser scanner in 3D mode is also performed for further investigations. An overview of the true trajectory is pictured in Figure 3 as a top view by means of the laser tracker measurements in two different scales for the y-axis.

### 3.2. Methodology

#### 3.2.1. Observation Vector

The local 2D LSP and 6D pose information by means of the laser tracker in combination with the T-Probe were already at hand by means of the sensor data available from the MSS mentioned in Section 3.1. The 6D pose observations of an IMU are simulated on the basis of such highly accurate reference pose information. For this purpose, noise and a linear drift were added to the reference given to model realistic observations of a moderate and an accurate IMU. This was obviously just a rough approximation and did not reproduce observations of an IMU in reality. However, additional influencing parameters were neglected for the sake of simplicity. Single or rather double integration over a time of 25 s (regarding the duration of measurements mentioned in Section 3.1) for angular velocity and acceleration stability was used. Based on this, it was assumed that a drift in the position of ∼16 m (moderate IMU) or rather ∼2.5 m (accurate IMU) and a drift in the orientation of ∼5${}^{\circ }$ (moderate IMU) or rather ∼0.2${}^{\circ }$ (accurate IMU) was acquired. Due to the lack of information perpendicular to the scanning plane of the laser scanner (in the direction of movement), the position information in the x-direction was not affected by these changes and was consistently equal to a respected reference. The sampling rate of the simulated IMU observations was identical to that of the reference data.

Afterwards, the observation vector $l_{k}$, consisting of one local LSP $P_{k}^{\mathrm{local}}$ (which consists, in turn, of *N* single 3D scan points), the 3D position $t_{k}$ and 3D rotation matrix $R_{k}$ (which is set up based on the three Euler angles $\Omega_{k}$, $\Phi_{k}$ and $K_{k}$) of the IMU are derived for each epoch k=1…K. Apart from that, the 6D reference pose of the laser tracker (position $t_{k}^{*}$ and rotation matrix $R_{k}^{*}$) could be relied directly on for the purpose of validation.

(23) $$\begin{array}{c} l_{k}={\left[\underset{P_{k}^{\mathrm{local}}}{\underset{︸}{x_{1,k},y_{1,k},z_{1,k},\ldots ,x_{N,k},y_{N,k},z_{N,k}}},\underset{t_{k}}{\underset{︸}{X_{k},Y_{k},Z_{k}}},\underset{R_{k}}{\underset{︸}{\Omega_{k},\Phi_{k},K_{k}}}\right]}^{T}. \end{array}$$

Additionally, the corresponding VCM $\Sigma_{\mathrm{vv}}$ of the observations $l_{k}$ can be set up, which consists of variances of the IMU $\Sigma_{{\mathrm{vv}}_{\mathrm{IMU}}}$ and the quality information of LSP in the form of variances $\Sigma_{{\mathrm{vv}}_{\mathrm{LSP}}}$. Related standard deviations for the VCM $\Sigma_{\mathrm{vv}}$ are given in Table 1. As has already been mentioned, IMU observations in the direction of movement (*x*) were assumed to be considerably more accurate than the ones in the perpendicular direction (*Y* and *Z*). Furthermore, the VCM $\Sigma_{{\mathrm{vv}}_{\mathrm{LSP}}}$ applied for LSP was not based directly on the range noise of the laser scanner given by the manufacture. It was concluded in the context of former investigations that such specifications were overoptimistic within the scope of the current approach proposed. This was due to the fact that the observations had to fulfil additional equations (e.g., geometrical constraints) and needed to be more variable. Consequently, standard deviations of the VCM $\Sigma_{{\mathrm{vv}}_{\mathrm{LSP}}}$ for LSP were larger than the manufacturer’s specifications and selected generously.

(24) $$\begin{array}{ccc} \Sigma_{{\mathrm{vv}}_{\mathrm{LSP}}} & = & diag\left(\sigma_{x_{1}}^{2},\sigma_{y_{1}}^{2},\sigma_{z_{1}}^{2},\ldots ,\sigma_{x_{N}}^{2},\sigma_{y_{N}}^{2},\sigma_{z_{N}}^{2}\right) \end{array}$$

(25) $$\begin{array}{ccc} \Sigma_{{\mathrm{vv}}_{\mathrm{IMU}}} & = & diag\left(\sigma_{X}^{2},\sigma_{Y}^{2},\sigma_{Z}^{2},\sigma_{\Omega }^{2},\sigma_{\Phi }^{2},\sigma_{K}^{2}\right) \end{array}$$

(26) $$\begin{array}{c} \Sigma_{\mathrm{vv}}=\left[\begin{array}{cc} \Sigma_{{\mathrm{vv}}_{\mathrm{LSP}}} & 0\\ 0 & \Sigma_{{\mathrm{vv}}_{\mathrm{IMU}}}. \end{array}\right] \end{array}$$

#### 3.2.2. Assignment Algorithm for Distinctive Planes

**Figure 4:**
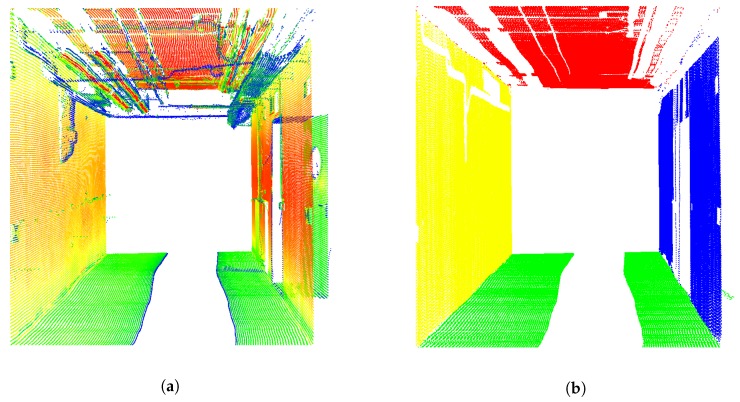
Georeferenced 3D point cloud of the environment measured based on the reference pose by means of laser tracker and T-Probe. Original scan points with colors by means of intensity (**a**). Assigned scan points regarding the left wall (yellow), right wall (blue), ceiling (red) and floor (green) (**b**).

Further indispensable information were the assignments of every single 3D scan point to distinctive planes (left wall, right wall, ceiling and floor) of the environment. For this purpose, every captured LSP $P_{k}^{\mathrm{local}}$ was segmented individually to identify the walls, ceiling and floor properly. This is done by a RANSAC algorithm in order to find suitable line segments within each single LSP. Applied distance threshold for the consensus set was 5 mm in combination with a maximum of 30 iterations. Suitable candidates have a minimum percentage (2%) of points in comparison to the total number of points within the respective LSP. Additionally, at least 20 points needed to be assigned to a line segment. In order to only identify lines, which represented left or right walls or rather ceilings or floors, only those candidates were selected which are almost parallel or perpendicular regarding the standing axis of the laser scanner (which is known by means of the local coordinate system). In order to avoid doors, leads or other obstacles, line candidates were compared regarding averaged assignments of several past LSPs (named as memory subsequently). The criteria used for this are changes in distance between the respective line and origin of the laser scanner and the variation of the averaged intensity of respective scan points. Both criteria are analyzed regarding the memory mentioned. Rough outliers in the assignment could be identified by applying such a restriction. Finally, every *N* single 3D scan point of the LSP $P_{k}^{\mathrm{local}}$ within each epoch k=1…K is assigned to left wall, right wall, ceiling, floor or remains as unused. These extended LSP are denoted $C_{k}^{\mathrm{local}}$ subsequently. Thus, in total, $C_{k}^{\mathrm{local}}$ is equal to $P_{k}^{\mathrm{local}}$ but contains mentioned additional segmentation information for every measured scan point. The results of the assignment algorithm introduced in relation to the case study are depicted in Figure 4a,b. Interfering objects (e.g., pipes and cables) are erased.

#### 3.2.3. Measurement Equation and State Parameter Vector

The state parameters desired were inter alia, relative changes in 3D position $\Delta t_{k}$, 3D orientation $\Delta R_{k}\left(\Delta \Omega_{k},\Delta \Phi_{k},\Delta K_{k}\right)$ and 3D velocity $\Delta v_{k}$. In combination with the (simulated) noisy and drifted IMU pose $\left(t_{k},R_{k}\right)$ they ended up in the almost true position $t_{k}^{\mathrm{MSS}}$ and orientation $R_{k}^{\mathrm{MSS}}\left(\Omega_{k}^{\mathrm{MSS}},\Phi_{k}^{\mathrm{MSS}},K_{k}^{\mathrm{MSS}}\right)$ of the MSS at each epoch k:

(27) $$\begin{array}{c} t_{k}^{\mathrm{MSS}}=t_{k}+\Delta t_{k},\;\;\;\;\;\;\;\;\;\;\;R_{k}^{\mathrm{MSS}}=\Delta R_{k}\cdot R_{k}. \end{array}$$

This formulation of relative changes as states might evoke a relation towards an error-state KF (ErKF, or indirect KF) [24,25]. However, the underlying concept of an ErKF is different. Instead of direct relative measurements (e.g., from an IMU), we included laser scanner observations by means of an implicit formulation. Georeferencing of every local LSP $P_{k}^{\mathrm{local}}$ regarding the superordinated coordinate system $P_{k}^{\mathrm{global}}$ can be applied by transformation using the estimated pose of the MSS:

(28) $$\begin{array}{c} P_{k}^{\mathrm{global}}=\left(t_{k}+\Delta t_{k}\right)+\left(\Delta R_{k}\cdot R_{k}\right)\cdot P_{k}^{\mathrm{local}}. \end{array}$$

At this point, prior information is integrated into our approach. Several geometrical circumstances could be taken into consideration during the movement through the corridor (cf. Figure 2b). In the current case, it is assumed that certain parts of all individual LSP’s captured some random parts of the left wall, right wall, ceiling and floor of the environment. Within a certain region, it could also be presumed that respective detected points on the left wall, right wall, ceiling and floor each refer to the same geometrical planes, respectively.

By using such information, the measurement equation could be formulated by means of the well-known Hesse normal form of a plane:

(29) $$\begin{array}{c} n_{e}\cdot \underset{C_{k}^{\mathrm{global}}}{\underset{︸}{\left[\left(t_{k}+\Delta t_{k}\right)+\left(\Delta R_{k}\cdot R_{k}\right)\cdot C_{k}^{\mathrm{local}}\right]}}-d_{e}=0, \end{array}$$

where $n_{e}$ is the 3×1 normal vector of the left wall (or rather right wall, ceiling, floor) and $d_{e}$ the related distance to the origin. Additionally, the segmented LSP information mentioned $C_{k}^{\mathrm{local}}$ regarding the left and right wall, ceiling and floor of the environment (cf. Section 3.2.2) could also be taken into consideration. In relation to Section 2.1 the given overall measurement Equation in (29) has an implicit formulation of type h(l,x)=0.

Hence, the 25-dimensional state parameter vector $x_{k}$ could be set up by means of the relative changes requested in position $\Delta t_{k}$, orientation $\Delta R_{k}\left(\Delta \Omega_{k},\Delta \Phi_{k},\Delta K_{k}\right)$ and velocity $\Delta v_{k}$ and four sets of plane parameters with each four parameters $n_{e_{x}},n_{e_{y}},n_{e_{z}},d_{e}$. Here, e can stand for the left wall (or rather right wall, ceiling or floor):

(30) $$\begin{array}{c} x_{k}={\left[\underset{\Delta t_{k}}{\underset{︸}{\Delta X_{k},\Delta Y_{k},\Delta Z_{k}}},\underset{\Delta R_{k}}{\underset{︸}{\Delta \Omega_{k},\Delta \Phi_{k},\Delta K_{k}}},\underset{\Delta v_{k}}{\underset{︸}{\Delta v_{x_{k}},\Delta v_{y_{k}},\Delta v_{z_{k}}}},\underset{leftwall}{\underset{︸}{n_{\zeta_{x}},n_{\zeta_{y}},n_{\zeta_{z}},d_{\zeta }}},\ldots ,\underset{floor}{\underset{︸}{n_{\xi_{x}},n_{\xi_{y}},n_{\xi_{z}},d_{\xi }}}\right]}^{T}. \end{array}$$

It is worth mentioning that the increase of epochs in trajectories is associated with the increase of geometric details (e.g., walls) of buildings in the environment within real world application. This leads to an unlimited expansion of the state vector. The usage of a dual state Kalman filter (DKF) in such a case might be suitable. This would enable strict separation of time changing states (e.g., position, orientation, velocity) and other over time static parameters (e.g., normal vector and distances to origin of a plane) [26]. However, interaction of DKF and implicit measurement equations was not treated in this paper.

#### 3.2.4. System Equation

A simple physical model was used to predict the constraint states from previous epoch k−1 to the current k. This state transition was based on a constant velocity model, which only affected the six pose parameters and three velocities of the state parameter vector [27]. All plane parameters were unaffected by this prediction step and were equal to the constraint state ${\tilde{x}}_{k-1}^{+}$:

(31) $$\begin{array}{cc} \Delta {\hat{t}}_{k}^{-}= & \Delta {\hat{t}}_{k-1}^{+}+\Delta {\hat{v}}_{k-1}^{+}\cdot \Delta \tau +w_{\Delta t,k-1} \end{array}$$

(32) $$\begin{array}{cc} \Delta {\hat{R}}_{k}^{-}= & \Delta {\hat{R}}_{k-1}^{+}+w_{\Delta R,k-1} \end{array}$$

(33) $$\begin{array}{cc} \Delta {\hat{v}}_{k}^{-}= & \Delta {\hat{v}}_{k-1}^{+}+w_{\Delta v,k-1} \end{array}$$

(34) $$\begin{array}{cc} {\hat{n}}_{e,k}^{-}= & {\hat{n}}_{e,k-1}^{+} \end{array}$$

(35) $$\begin{array}{cc} {\hat{d}}_{e,k}^{-}= & {\hat{d}}_{e,k-1}^{+}, \end{array}$$

where $w_{\Delta t,k-1}$, $w_{\Delta R,k-1}$ and $w_{\Delta v,k-1}$ are the process noise vectors and Δτ is the time interval between two consecutive epochs. The VCM of the process noise $\Sigma_{\mathrm{ww}}$ represents related system noise during the prediction step. Due to simplicity, all variances and covariances were zero, except for the process noise of the velocity. Within this case study a definition of $\sigma_{v,w}=5\cdot \Delta \tau$ is selected.

(36) $$\begin{array}{c} \Sigma_{\mathrm{ww}}=\left[\begin{array}{cc} diag\left(0_{\left[1\times 6\right]},\right.\sigma_{v,w}^{2},\sigma_{v,w}^{2},\sigma_{v,w}^{2} & 0\\ 0 & diag\left(0_{\left[1\times 16\right]}\right) \end{array}\right]. \end{array}$$

#### 3.2.5. Nonlinear Equality and Inequality Constraint for the State Parameters

In addition to the measurement Equation (29), the geometric prior information by means of equality and inequality constraints is also used to improve the georeferencing of the MSS. Due to the fact that the plane parameters $n_{e}$ within the state parameter vector $x_{k}$ were used, the unity of normal vectors had to be ensured. In order to do so, nonlinear equality constraints can be used:

(37) $$\begin{array}{c} g\left(x_{k}\right)=\left|\right|n_{e}\left|\right|=\sqrt{n_{e_{x}}^{2}+n_{e_{y}}^{2}+n_{e_{z}}^{2}}=b=1. \end{array}$$

Furthermore, inequality constraints regarding intersection angles of related planes are also implemented. In this context, obvious conditions for concurrency and perpendicularity between distinctive walls are relied on. It would also be possible to formulate these constraints by means of equality constraints. However, instead of using such hard constraints, the use of inequality constraints together with lower ${\mathrm{lb}}_{i,k}$ and upper ${\mathrm{ub}}_{i,k}$ boundaries (cf. Section 2.2) are preferred. Applying such inequality constraints is more consistent with reality, where such perfect conditions are rather rare or can be rarely fulfilled. Selected thresholds for this are derived based on documented standards for the building industry [28] and should be stated around $0^{\circ }$ (for concurrency) and $90^{\circ }$ (for perpendicularity). As a further basis, the information based on the highly accurate static 3D laser scanner point cloud mentioned are used to set up the boundaries. By means of this reference, true intersection angles between walls can be determined and applied. Consequently, the boundaries are selected by considering $0.5^{\circ }$ for the intersection angles mentioned:

(38) $$\begin{array}{c} g_{i}\left(x_{k}\right)=cos^{-1}\left(\frac{|n_{\zeta }\cdot n_{\xi }|}{|n_{\zeta }\left|\cdot \right|n_{\xi }|}\right)=cos^{-1}\left(\frac{|n_{\zeta_{x}}n_{\xi_{x}}+n_{\zeta_{y}}n_{\xi_{y}}+n_{\zeta_{z}}n_{\xi_{z}}|}{\sqrt{n_{\zeta_{x}}^{2}+n_{\zeta_{y}}^{2}+n_{\zeta_{z}}^{2}}\cdot \sqrt{n_{\xi_{x}}^{2}+n_{\xi_{y}}^{2}+n_{\xi_{z}}^{2}}}\right) \end{array}$$

(39) $$\begin{array}{c} {\mathrm{lb}}_{i,k}\leq g_{i}\left(x_{k}\right)\leq {\mathrm{ub}}_{i,k}\;\;\;\;\;\;\;\;\;\;\mathrm{with}:\;\;\;{\mathrm{lb}}_{i,k}=g_{i}\left(x_{k}\right)-0.5^{\circ },\;\;\;\;{\mathrm{ub}}_{i,k}=g_{i}\left(x_{k}\right)+0.5^{\circ }. \end{array}$$

Due to the geometrical behaviour of the environment, there are several possibilities to apply Equation (38) in terms of concurrency or rather perpendicularity. For this reason, there are also different options for the number of geometrical inequality constraints selected. Within Section 3.3, the respective impacts and benefits of combining several constraints in contrast to individual use cases are shown. Regardless of the respective combination, which constraints are active within each epoch should be checked. This means that constraints can only be applied if at least five points of the related walls, ceiling or floor are segmented within this epoch. If there is a lack of one or several walls, all respective constraints to this wall will be inactive for this epoch.

#### 3.2.6. Initialization

Initialization of approximate values for the state vector $x_{0}$ and the related VCM $\Sigma_{\mathrm{xx},0}$ are needed to perform the IEKF. Initial relative changes in position $\Delta t_{0}$ and orientation $\Delta R_{0}$ are selected by means of the difference between the reference pose and IMU regarding first epoch k=0. Relative velocities $\Delta v_{0}$ are initialized as zero. Initial values for the normal vectors of the planes $n_{e,0}$, are estimated by means of the first LSP and its respective points for left wall, right wall, ceiling and floor. Related standard deviations for the VCM $\Sigma_{\mathrm{xx},0}$ are given in Table 2.

(40) $$\begin{array}{c} x_{0}={\left[\Delta X_{0},\Delta Y_{0},\Delta Z_{0},\Delta \Omega_{0},\Delta \Phi_{0},\Delta K_{0},\Delta v_{x_{0}},\Delta v_{y_{0}},\Delta v_{z_{0}},n_{\zeta_{x},0},n_{\zeta_{y},0},n_{\zeta_{z},0},d_{\zeta ,0},\ldots ,n_{\xi_{x},0},n_{\xi_{y},0},n_{\xi_{z},0},d_{\xi ,0}\right]}^{T} \end{array}$$

(41) $$\begin{array}{c} \Sigma_{\mathrm{xx},0}=diag\left(\sigma_{\Delta X}^{2},\sigma_{\Delta Y}^{2},\sigma_{\Delta Z}^{2},\sigma_{\Delta \Omega }^{2},\sigma_{\Delta \Phi }^{2},\sigma_{\Delta K}^{2},\sigma_{\Delta v_{x}}^{2},\sigma_{\Delta v_{y}}^{2},\sigma_{\Delta v_{z}}^{2},\sigma_{n_{\zeta_{x}}}^{2},\sigma_{n_{\zeta_{y}}}^{2},\sigma_{n_{\zeta_{z}}}^{2},\sigma_{d_{\zeta }}^{2},\ldots ,\sigma_{n_{\xi_{x}}}^{2},\sigma_{n_{\xi_{y}}}^{2},\sigma_{n_{\xi_{z}}}^{2},\sigma_{d_{\xi }}^{2}\right). \end{array}$$

### 3.3. Results

**Figure 5:**
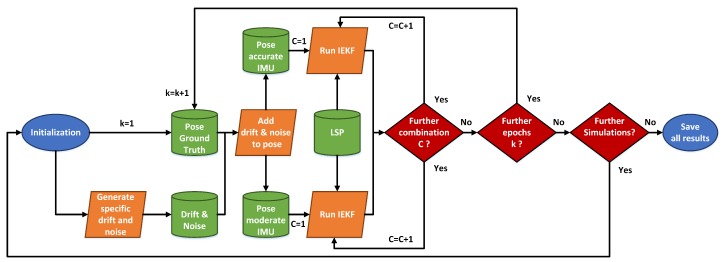
Schematic overview of the 500 replications performed for two types of IMUs as required input data for the iterated extended Kalman filter (IEKF) from Section 2.1 and its related combination C=I…X of applied state constraints. The Roman numerals refer to respective state constraints applied regarding Table 3.

In order to ensure independence from simulated IMU pose information, the results within this Section 3.3 are, with respect to the mean of 500 replications, of slightly different realizations of the IMU pose information. Additionally, to investigate the differences with respect to a moderate and an accurate IMU, results of two sets of simulations are presented within this Section 3.3. A schematic overview of this procedure is depicted in Figure 5. Evaluation is done by means of the estimated pose parameters of the kinematic MSS $t_{k}^{\mathrm{MSS}}$ and $R_{k}^{\mathrm{MSS}}$ and the ground truth by means of the laser tracker $t_{k}^{\mathrm{GT}}$ and $R_{k}^{\mathrm{GT}}$. Based on these pose information, the root mean square error (RMSE) for the combined position in the x-, y-, z-direction can be calculated (cf. (42)). In order to give a quality parameter for combined orientation, transformation from the rotation matrix $R_{k}$ to the axis-angle representation by a normalized vector $r_{k}=\left[r_{1},r_{2},r_{3}\right]$ and rotation angle $\Theta_{k}$ is performed. Afterwards, the mean error (ME) of the representative angle between estimation $\Theta_{k}^{\mathrm{MSS}}$ and ground truth $\Theta_{k}^{\mathrm{GT}}$ is calculated and used (cf. (43)). Presentation of the results by means of combined position and orientation instead of a single axis is intended. In such a manner, we can identify the most suitable combination of state constraints for this approach while keeping the results clear.

(42) $$\begin{array}{ccc} RMSE & = & \sqrt{\frac{1}{k}\sum_{i=1}^{k}\left({\left(X_{k}^{\mathrm{GT}}-X_{k}^{\mathrm{MSS}}\right)}^{2}+{\left(Y_{k}^{\mathrm{GT}}-Y_{k}^{\mathrm{MSS}}\right)}^{2}+{\left(Z_{k}^{\mathrm{GT}}-Z_{k}^{\mathrm{MSS}}\right)}^{2}\right)} \end{array}$$

(43) $$\begin{array}{ccc} ME & = & \frac{1}{k}\sum_{i=1}^{k}\left(|\Theta_{k}^{\mathrm{GT}}-\Theta_{k}^{\mathrm{MSS}}|\right). \end{array}$$

**Figure 6:**
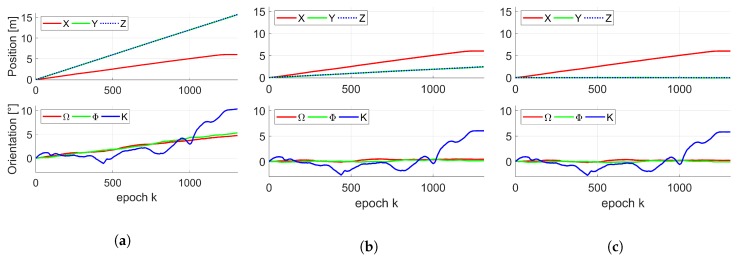
Mean change in position (top) and orientation (bottom) of the kinematic MSS by means of 500 simulated moderate IMU poses (**a**) and accurate IMU poses (**b**) over K epochs. True change in position (top) and orientation (bottom) of the kinematic MSS by means of laser tracker pose (**c**) over K epochs.

The difference between both classes of IMUs, as well as ground truth, is shown in Figure 6 by means of their averaged change in position and orientation over all corresponding 500 replications. As it has already been mentioned in Section 3.2.1, position in the x-direction for both IMUs was identical to the ground truth by means of the laser tracker. However, a major linear drift is visible (∼15 m for moderate IMU or rather ∼2.5 m for accurate IMU) for position in both other directions. Due to different assumed uncertainties for both IMUs, the drift in orientation for the accurate IMU was rather negligible, whereas the drift for the moderate IMU was about ∼5${}^{\circ }$ for all axes.

Based on this IMU pose information, methods from Section 3.2 are applied. In addition to both classes of IMUs, combinations of respective equality and inequality state constraints also affects the pose parameters requested. In the framework of this paper, estimates of ten different combinations (listed in Table 3) are presented. In order to compare various constraints in the developed IEKF algorithm, the combination I was designed without using any constraints and will be considered as a reference solution. All other combinations (II–X) relied on different equality and inequality constraints which include concurrency and/or perpendicularity between assigned left/right wall or rather ceiling and floor. In all these combinations the constraints regarding normal plane vectors (cf. (37)) were formulated in order to ensure numerical and geometrical stability. The inequality constraints in combinations III–VI were applied independently from each other whereas in combination VII–X a collaboration between concurrency and perpendicularity was enabled in order to evaluate respective impact on the state estimates for each collaboration. However, it is worth mentioning that the impact of the individual combinations might vary depending on respective application and related environmental circumstances.

The results achieved over all 500 replications for both moderate and accurate IMU observations in relation to the ten different combinations of applied state constraints are summarized in Table 4 for combined position by means of RMSE and Table 5 for combined orientation by means of ME. Comparison between the results was determined by means of minimum (min), maximum (max), mean, median and standard deviation (SD), as well as lower bound (↓) and upper bound (↑) of the 95% confidence interval (CI), calculated numerically from the 500 samples, as selected characteristic values.

It is notable that, independently from the IMU used, pose estimation fails if no constraints (combination I) are applied. It further stands out that there was an impact of the RMSE and ME depending on the constraints applied. In terms of position, combination III delivers the lowest estimates for both IMUs. Whereas for the moderate IMU, combination X was the lowest in terms of orientation, and for the accurate IMU, combination III is also the lowest (both judged by median). However, without taking into account combination I, all solutions by means of the applied state constraints were smaller than the ME for orientation of the moderate IMU. For the accurate IMU only combinations III, V, VI and VIII were smaller than the noisy and drifted IMU solution. However, the gain in accuracy is much higher for the position compared to the orientation.

**Figure 7:**
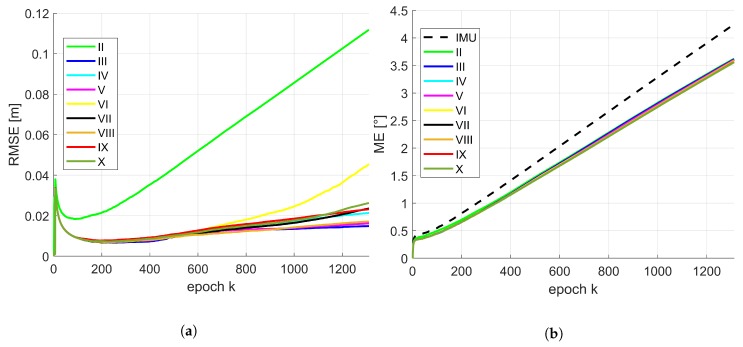
Moderate IMU: temporal progress of the median of the root mean square error (RMSE) for position (**a**) and mean error (ME) for orientation (**b**) by means of 500 replications for respective combinations of the state constraints applied. The Roman numerals refer to respective state constraints applied regarding Table 3.

**Figure 8:**
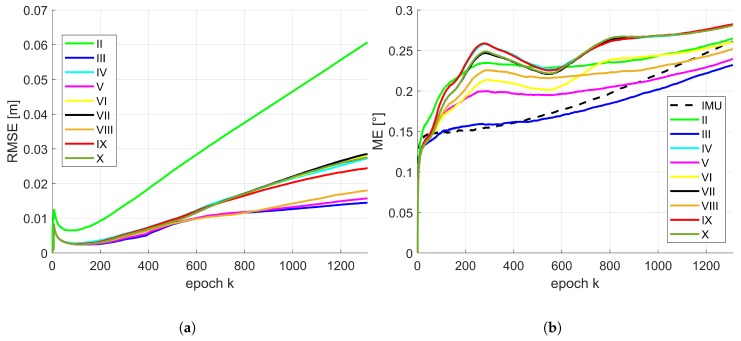
Accurate IMU: temporal progress of the median of the RMSE for position (**a**) and ME for orientation (**b**) by means of 500 replications for respective combinations of the state constraints applied. The Roman numerals refer to respective state constraints applied regarding Table 3.

Due to the conclusions provided by means of Table 4 and Table 5, the temporal progress in position and orientation of the RMSE or rather ME for different combinations of state constraints are depicted in Figure 7 for the moderate IMU. The same results regarding the accurate IMU are depicted in Figure 8. For presentation purposes, inaccurate solutions (e.g., IMU in terms of position; combination I) are omitted. The basic behaviour of the temporal progress of the RMSE for position of both IMUs was very similar. All combinations increased drastically within the very first epochs. After this running-in effect of the filter they decrease quickly and continue differently over time. Over all epochs, combination II leads to a significant larger RMSE and has the largest increase. This is of interest, except normalized plane normal vectors, no further geometrical constraints like concurrency or perpendicularity were considered within this configuration. Combination III, V and VIII are very similar and lead to the best results around 1.5 cm. Remaining combinations have a slightly larger increase and will end up between 2–5 cm. Temporal behaviour of the ME for orientation is slightly different for both IMUs. They also increased drastically within the very first epochs. Afterwards, the gradient was related to the initially drifted IMU solution. However, all presented solutions for the moderate IMU were lower than respective initial IMU solution. In addition, gradient and progress are almost identical for this type of IMU. In case of the accurate IMU, there was a slight variation between all combinations. But from epoch *k* = 800 the increase was for all combinations lower than the IMU solution. Combination III behaves most similar to the IMU solution in the beginning and undercut the IMU curve at epoch *k* = 400.

**Figure 9:**
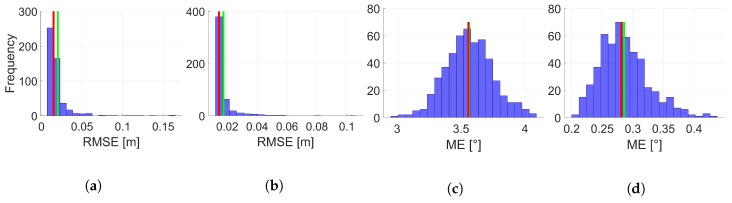
Histograms of the RMSE for position by means of 500 replications for combination III (moderate IMU (**a**)) and combination III (accurate IMU (**b**)) of state constraints applied. Related histograms of the ME for orientation by means of 500 replications for combination X (moderate IMU (**c**)) and combination III (accurate IMU (**d**)) of state constraints applied. Respective mean is given by a red bar and respective median by a green bar. The Roman numerals refer to respective state constraints applied regarding Table 3.

In order to investigate the individual best results for position and orientation, respective histograms regarding the related 500 replications are depicted in Figure 9 for the moderate and accurate IMU with respect to each other. Based on these representations, further conclusions can be drawn. All histograms show distributions which are right-skew symmetric. This indicates that, independent from the IMU observation applied, there are a few configurations within the respective 500 replications which lead to a slightly larger RMSE or rather ME or even outliers. However, this skew is much more pronounced in case of the RMSE for position. The histograms for the ME of the orientation are similar to a Gaussian distribution. This different behaviour is not directly explainable and further investigations are needed. For this reason, a more detailed arrangement regarding the single coordinate axis in contrast to the combined presentation appears appropriate and will be realized in the future.

In general, it can be summarized that the consideration of state constraints improved state estimation significantly. However, differences between individual combinations were quite small. For this reason, geometrical restrictions regarding perpendicularity and concurrency depend strongly on respective environments.

## 4. Discussion

The results presented in Section 3.3 indicate significant dependencies of the estimated pose parameters on the respective equality and inequality state constraints applied. Moreover, no prominent combination of constraints exists which fits to all requirements in terms of position and orientation. The two different types of IMU observations demonstrate additional dependencies. Depending on the respective accuracy class, the use of certain constraints can significantly improve pose estimation. This applies particularly to the orientation estimation, whereas position estimation benefits from almost every constraint applied, although to different levels. Overall, it could be seen that the usage of state constraints results in an important added value. However, there is an important need to define and apply a suitable model selection procedure into the current filter approach. Various different constraint combinations are applied and such a procedure should determine which combination is most suitable. Our priors will be obtained by considering which constraints are representative of features we expect to see in the data, and which would produce biased or inaccurate estimates. In addition, the effects of different individual constraints, in contrast to combined constraints, will be investigated more extensively in the future. Possible linear dependencies between individual constraints need to be analyzed and, if necessary, neglected.

As it has already been mentioned in Section 2.1, the compatibility of implicit measurement equations and state constraints as part of the IEKF is an important issue which needs further consideration. Due to implicit formulation, both states and observations are corrected within the update step to fulfill the measurement equation. During the constraint step, only the states are affected, while observations are unaffected. Consequently, this leads to a violated measurement equation. For this reason, the constraint step is going to be directly integrated into the update step of the IEKF. In terms of equality constraints, extension of the objective function should be sufficient. However, in terms of inequality constraints, this is not possible straightforwardly. Combination of inequality state constraints in the scope of GHM is treated in [29] and shows the complexity relating thereto. Another more promising approach for this task will be to use soft constraints instead of inequality constraints, which will be investigated in the future. Also, consideration of perfect measurements is possible, however, this would only allow equality constraints. In theory, state constraints can also be applied in combination with other classes of Kalman filters (e.g., UKF) to avoid the factual linearization issues of the EKF and IEKF. However, compatibility of implicit measurement equations besides linear KF, EKF and IEKF need to be solved first.

The dependency mentioned regarding the IMU observations applied is an argument to consider initial biases and drift parameters of the IMU as additional state parameters within further developments. By doing so, direct estimation and consideration within the IEKF are possible and should further enhance the pose estimation. As a consequence of such an extension, this would be accompanied by further development of a more suitable system model within the prediction step.

## 5. Conclusions

We presented a novel method to consider nonlinear equality and inequality state constraints within the framework of an IEKF with implicit measurement equations. Consideration of such restrictions is realized by means of flexible PDF truncation. This method was applied and evaluated for georeferencing of an indoor laser scanner-based kinematic MSS. Therefor, different combinations of geometrical constraints were applied for real measurement data.

In conclusion, the consideration of appropriate restrictions between the state parameters is desirable. The use of inequality constraints in addition to equality constraints offers further possibilities in terms of accuracy. This justifies general consideration of inequality state constraints for georeferencing of a kinematic MSS.

Furthermore, adaptation and application of the general georeferencing approach by means of an IEKF with respect to other kinematic MSS is planned to verify its general validity. The focus there is to apply the approach on an UAV and an outdoor mobile mapping system. Both applications require special demands concerning 3D point cloud assignments in terms of facades, building models and further external influences which may occur within outdoor environments. In addition, the approach presented in this article has to be applied for longer data sets (with respect to spatial and temporal expansion). For this, it is assumed that the RMSE and ME will increase slightly over time unless absolute landmarks are integrated at certain points in time or assumed geometrical constraints are not applicable. Also nonlinear trajectories with turning manoeuvres need to be considered. This might make it necessary to introduce new planes and respective parameters into the model. However, applicability will be ensured as long as sufficient additional information from object space are available, assignable and applicable.

In addition, a more simple application example might be suitable to evaluate a comparison of different constraint combinations and methods to apply them to the fundamental algorithm of the IEKF with implicit measurement equations. As mentioned in Section 2, also other methods can be applied to consider state constraints. While PDF truncation provides great flexibility in simultaneously applying equality and inequality constraints, other methods mentioned above may be more appropriate (e.g., with respect to the uncertainty of the estimated state parameters and the computing time of the algorithm). However, methods for inequality constraints are limited as long as quadratic programming problems should be avoided.

## Figures and Tables

**Table 1:** Scheduled standard deviations σ for the variance-covariance matrix (VCM) $\Sigma_{\mathrm{vv}}$ of the observation vector $l_{k}$.

Sensor Type	Observation	Assumed σ	
		Moderate IMU	Accurate IMU
Laser scanner	x,y,z	3 mm	3 mm
IMU	*X*	0.01 mm	0.01 mm
	Y,Z	80 mm	20 mm
	Ω,Φ,K	0.2${}^{\circ }$	0.07${}^{\circ }$

**Table 2:** Scheduled standard deviations σ for the initial VCM $\Sigma_{\mathrm{xx},0}$ of the initial state vector $x_{0}$.

State Parameter	σ
ΔX,ΔY,ΔZ	0.1 m
ΔΩ,ΔΦ,ΔK	$5.7^{\circ }$
$\Delta v_{x},\Delta v_{y},\Delta v_{z}$	0.1 m/s
$n_{e_{x}},n_{e_{y}},n_{e_{z}}$	0.1
$d_{e}$	0.1 m

**Table 3:** Investigated combinations of respective equality (red) and inequality (green) state constraints. Applied constraints within each combination are depicted with a ✓ symbol.

	Combinations of Respective Equality and Inequality State Constraints									
	**I**	**II**	**III**	**IV**	**V**	**VI**	**VII**	**VIII**	**IX**	**X**
unit vector for left wall		✓	✓	✓	✓	✓	✓	✓	✓	✓
unit vector for right wall		✓	✓	✓	✓	✓	✓	✓	✓	✓
unit vector for ceiling		✓	✓	✓	✓	✓	✓	✓	✓	✓
unit vector for floor		✓	✓	✓	✓	✓	✓	✓	✓	✓
left/right wall are parallel			✓				✓	✓		✓
ceiling/floor are parallel				✓			✓		✓	✓
left wall/ceiling are perpendicular					✓			✓	✓	✓
right wall/floor are perpendicular						✓				✓

**Table 4:** Root mean square error (RMSE) for position by means of 500 replications. The Roman numerals refer to respective state constraints applied regarding Table 3. Each of the seven characteristic values (minimum, maximum, mean, median, standard deviation (SD) as well as lower bound (↓) and upper bound (↑) of the 95% confidence interval (CI)) are divided into two additional rows regarding moderate (above) and accurate (below) inertial measurement unit (IMU). The largest (red) and lowest (green) estimates are marked for first five rows.

		Combinations of Respective Equality and Inequality State Constraints									
	**IMU**	**I**	**II**	**III**	**IV**	**V**	**VI**	**VII**	**VIII**	**IX**	**X**
Min [m]	12.646 1.9833	1.4684 0.8992	0.0118 0.0114	0.0128 0.0140	0.0147 0.0168	0.0130 0.0142	0.0135 0.0143	0.0161 0.0209	0.0130 0.0142	0.0168 0.0159	0.0164 0.0209
Max [m]	13.030 2.0864	3469.2 2.4$\cdot 10^{5}$	5.7065 4.3490	0.1649 0.1049	0.2269 0.1425	0.1360 0.0561	0.2460 0.1122	0.1234 0.0966	0.2278 0.0781	0.4138 0.2269	141.08 0.0699
Mean [m]	12.835 2.0336	79.778 1974.7	0.3188 0.1828	0.0201 0.0174	0.0280 0.0312	0.0218 0.0182	0.0470 0.0327	0.0269 0.0304	0.0226 0.0206	0.0335 0.0282	0.3139 0.0291
Median [m]	12.832 2.0337	9.9179 8.8968	0.1118 0.0607	0.0149 0.0145	0.0214 0.0273	0.0162 0.0157	0.0455 0.0284	0.0236 0.0286	0.0172 0.0180	0.0232 0.0244	0.0263 0.0275
SD [m]	0.0678 0.0176	320.29 16083	0.6136 0.3384	0.0160 0.0080	0.0192 0.0130	0.0151 0.0064	0.0272 0.0167	0.0116 0.0081	0.0163 0.0075	0.0325 0.0147	6.3077 0.0063
↓95% CI [m]	12.714 1.9983	2.7926 2.0866	0.0189 0.0137	0.0134 0.0141	0.0163 0.0194	0.0133 0.0143	0.0140 0.0146	0.0171 0.0236	0.0135 0.0144	0.0179 0.0181	0.0178 0.0227
↑95% CI [m]	12.960 2.0707	559.22 7126.2	1.9255 0.9893	0.0596 0.0405	0.0742 0.0647	0.0729 0.0409	0.1042 0.0771	0.0563 0.0525	0.0654 0.0414	0.1155 0.0570	0.0947 0.0498

**Table 5:** Mean error (ME) for orientation by means of 500 replications. The Roman numerals refer to respective state constraints applied regarding Table 3. Each of the seven characteristic values (minimum, maximum, mean, median, SD as well as lower bound (↓) and upper bound (↑) of the 95% CI) are divided into two additional rows regarding moderate (above) and accurate (below) IMU. The largest (red) and lowest (green) estimates are marked for first five rows.

		Combinations of Respective Equality and Inequality State Constraints									
	**IMU**	**I**	**II**	**III**	**IV**	**V**	**VI**	**VII**	**VIII**	**IX**	**X**
Min [°]	3.6742 0.1548	4.6565 2.1602	2.9283 0.1522	2.9843 0.1541	2.9558 0.1986	3.0133 0.1557	2.9283 0.1498	2.9544 0.2151	2.9793 0.1535	2.9560 0.2084	2.9503 0.2100
Max [°]	4.8145 0.4367	42.297 32.819	4.3253 0.4542	4.1203 0.4114	4.0770 0.4455	4.0935 0.4197	4.0896 0.4218	4.0788 0.4434	4.0887 0.4279	4.0779 0.4460	4.0766 0.4369
Mean [°]	4.2414 0.2654	11.222 9.4379	3.6246 0.2727	3.6140 0.2371	3.5657 0.2864	3.5990 0.2447	3.5691 0.2628	3.5653 0.2860	3.5873 0.2562	3.5643 0.2857	3.5625 0.2856
Median [°]	4.2471 0.2611	10.355 8.8777	3.6242 0.2649	3.6117 0.2322	3.5619 0.2821	3.5992 0.2397	3.5689 0.2600	3.5619 0.2822	3.5874 0.2520	3.5615 0.2825	3.5568 0.2809
SD [°]	0.1890 0.0529	4.2427 4.1022	0.2302 0.0598	0.1807 0.0463	0.1863 0.0400	0.1814 0.0463	0.1893 0.0482	0.1871 0.0405	0.1838 0.0453	0.1866 0.0400	0.1874 0.0400
↓95% CI [m]	3.8684 0.1793	5.7887 3.9401	3.1598 0.1762	3.2611 0.1667	3.1979 0.2212	3.2463 0.1668	3.1921 0.1706	3.1992 0.2216	3.2241 0.1836	3.1982 0.2226	3.1970 0.2206
↑95% CI [m]	4.6200 0.3758	22.2408 19.2219	4.0898 0.4079	3.9940 0.3426	3.9578 0.3809	3.9841 0.3443	3.9604 0.3699	3.9565 0.3787	3.9702 0.3612	3.9562 0.3822	3.9542 0.3804
